# Supplementary material for: More homogeneous capillary flow and oxygenation in deeper cortical layers correlate with increased oxygen extraction
Source: eLife. 2019 Jul 15;8:e42299. doi: 10.7554/eLife.42299 (PMC6636997; doi:10.7554/eLife.42299)
Supplement: Supplementary file 1. [file elife-42299-supp1.docx]

**Supplementary Table 1.** Measurement information for the main analysis in Figs. 2-7.

| Parameters | Depth | Numbers of samples/mice | | |
| --- | --- | --- | --- | --- |
|  |  | **Capillaries** | **Arterioles** | **Venules** |
| Mean-PO_2_ | 0-600 µm | 978/15 | 11/7 | 14/7 |
| RBC Flux | 0-600 µm | 978/15 | N.A. | N.A. |
| Temporal Fluctuation | 0-600 µm | 373/7 | N.A. | N.A. |
| EATs | 0-600 µm | 373/7 | N.A. | N.A. |
| Branching Orders | 0-300 µm | 97/5 | N.A. | N.A. |
