# Supplementary material for: More homogeneous capillary flow and oxygenation in deeper cortical layers correlate with increased oxygen extraction
Source: eLife. 2019 Jul 15;8:e42299. doi: 10.7554/eLife.42299 (PMC6636997; doi:10.7554/eLife.42299)
Supplement: Transparent reporting form [file elife-42299-transrepform.pdf]

eLIFE
1st Floor
24 Hills Road
Cambridge CB2 1JP, UK
P 01223 855340
W elifesciences.org
T @elife

eLife's transparent reporting form

We encourage authors to provide detailed information *within their submission* to facilitate the interpretation and replication of experiments. Authors can upload supporting documentation to indicate the use of appropriate reporting guidelines for health-related research (see EQUATOR Network), life science research (see the BioSharing Information Resource), or the ARRIVE guidelines for reporting work involving animal research. Where applicable, authors should refer to any relevant reporting standards documents in this form.

If you have any questions, please consult our Journal Policies and/or contact us: editorial@elifesciences.org.

Sample-size estimation

- You should state whether an appropriate sample size was computed when the study was being designed
- You should state the statistical method of sample size computation and any required assumptions
- If no explicit power analysis was used, you should describe how you decided what sample (replicate) size (number) to use

Please outline where this information can be found within the submission (e.g., sections or figure legends), or explain why this information doesn't apply to your submission:

This information can be found in Materials and methods, subsection Statistical analysis.

Replicates

- You should report how often each experiment was performed
- You should include a definition of biological versus technical replication
- The data obtained should be provided and sufficient information should be provided to indicate the number of independent biological and/or technical replicates
- If you encountered any outliers, you should describe how these were handled
- Criteria for exclusion/inclusion of data should be clearly stated
- High-throughput sequence data should be uploaded before submission, with a private link for reviewers provided (these are available from both GEO and ArrayExpress)

Please outline where this information can be found within the submission (e.g., sections or figure legends), or explain why this information doesn't apply to your submission:

eLife Sciences Publications, Ltd is a limited liability non-profit non-stock corporation incorporated in the State of Delaware, USA, with company number 5030732, and is registered in the UK with company number FC030576 and branch number BR015634 at the address 1st Floor, 24 Hills Road, Cambridge CB2 1JP | August 2014

In each mouse, we performed two separate imaging experiments. One experiment was for acquiring the 3D angiograms, another was for acquiring PO_2 and capillary RBC flux. These two measurements were performed on two separate days, typically 2 days apart. This information was provided in Materials and methods, subsection Acquisition of microvascular angiograms. In each mouse, measurements (e.g., acquisition of angiogram or PO_2/RBC flux) was performed only once.

Biological replication: We typically performed one PO_2 and/or RBC flux acquisition per vessel segment. No replication was done at the level of individual vessels. Please see Results, subsection Accumulated oxygen extraction fraction increases in deeper cortical layers, line 120, and Materials and methods, subsection Intravascular PO2 imaging. Number of animals used and measured vascular segments were also reported in corresponding figure legends.

Technical replication: we repeated 2,000 or 30,000 two-photon excitation/decay cycles at each measurement location to obtain an average phosphorescence decay. Please see Materials and methods, subsections Intravascular PO_2 imaging, and Imaging of EATs and capillary RBC flow.

The data was excluded mostly due to the mouse motion artefacts. Please see Materials and methods, subsection Rejection of motion artefacts. In addition, 3 mice were not used in experiments because incomplete healing of the brain tissue after installation of the cranial window.

eLife Sciences Publications, Ltd is a limited liability non-profit non-stock corporation incorporated in the State of Delaware, USA, with company number 5030732, and is registered in the UK with company number FC030576 and branch number BR015634 at the address 1st Floor, 24 Hills Road, Cambridge CB2 1JP | August 2014

Statistical reporting

- Statistical analysis methods should be described and justified
- Raw data should be presented in figures whenever informative to do so (typically when N per group is less than 10)
- For each experiment, you should identify the statistical tests used, exact values of N, definitions of center, methods of multiple test correction, and dispersion and precision measures (e.g., mean, median, SD, SEM, confidence intervals; and, for the major substantive results, a measure of effect size (e.g., Pearson's r, Cohen's d)
- Report exact p-values wherever possible alongside the summary statistics and 95% confidence intervals. These should be reported for all key questions and not only when the p-value is less than 0.05.

Please outline where this information can be found within the submission (e.g., sections or figure legends), or explain why this information doesn't apply to your submission:

Statistical analysis method was described in Materials and Methods, subsection Statistical analysis. Statistical information was also reported in the figure legends, where relevant.

(For large datasets, or papers with a very large number of statistical tests, you may upload a single table file with tests, Ns, etc., with reference to sections in the manuscript.)

Group allocation

- Indicate how samples were allocated into experimental groups (in the case of clinical studies, please specify allocation to treatment method); if randomization was used, please also state if restricted randomization was applied
- Indicate if masking was used during group allocation, data collection and/or data analysis

Please outline where this information can be found within the submission (e.g., sections or figure legends), or explain why this information doesn't apply to your submission:

This information doesn't apply to our study. We used normal healthy C57BL/6 mice and explored resting state brain oxygen delivery. Our study was conducted with only one group consisting of n = 15 mice.

Additional data files ("source data")

- We encourage you to upload relevant additional data files, such as numerical data that are represented as a graph in a figure, or as a summary table
- Where provided, these should be in the most useful format, and they can be uploaded as "Source data" files linked to a main figure or table
- Include model definition files including the full list of parameters used
- Include code used for data analysis (e.g., R, MatLab)
- Avoid stating that data files are "available upon request"

Please indicate the figures or tables for which source data files have been provided:

We did not provide any source files at this point, but we would like to provide a summary data file at a later stage.
